# Impact of caffeic acid on the testicular damages in D-galactose-induced aging model in mice

**DOI:** 10.22038/IJBMS.2022.63977.14092

**Published:** 2022-10

**Authors:** Fatemeh Khoshdel, Mohammad Ghasem Golmohammadi, Mohammad Jannat Dost, Nowruz Najafzade, Ramin Salimnejad

**Affiliations:** 1 Department of Anatomical Sciences, School of Medicine, Ardabil University of Medical Sciences, Ardabil, Iran; 2 School of Medicine, Ardabil University of Medical Sciences, Ardabil, Iran

**Keywords:** Aging, Caffeic acid, D-galactose, Oxidative stress, Spermatogenesis

## Abstract

**Objective(s):**

Aging is a biological phenomenon that causes various disorders and diseases in body systems such as the reproductive system. One of the important factors in aging is oxidative stress, which facilitates the aging process through various mechanisms. The aim of this study is the investigation of effects of caffeic acid on the testicular damages in Dgalactose induced aging model in mice.

**Materials and Methods:**

Forty male mice were randomly divided into 5 groups (n=8): 1) Control, 2) Sham, 3) Aging, 4) Aging + caffeic acid, and 5) Caffeic acid. Aging was induced through daily injection of D-Galactose (300 mg/kg, intraperitoneal) for 6 weeks. Caffeic acid (60 mg/kg, intraperitoneal) was injected daily for 6 weeks. One day after the last injection mice were killed and the testicle and epididymis were removed. Then, sperm parameters, factors of oxidative stress, and histopathological changes were evaluated.

**Results:**

The results showed that aging significantly decreased the count, motility, and viability of sperm, and increased abnormal sperm and sperm DNA fragmentation in contrast to the control group (*P*<0.05). In addition, MDA levels increased significantly in this group, and SOD, GPx, and TAC activity decreased (*P*<0.05). Histological studies also showed the destruction of seminiferous tubules, and Johnson’s score decreased (*P*<0.05). Caffeic acid administration significantly improved the above disarrays (*P*<0.05).

**Conclusion:**

The results showed that caffeic acid reduces the adverse effects of aging on spermatogenesis in mice by reducing oxidative stress and increasing antioxidant defenses.

## Introduction

Aging is a biological phenomenon that causes various disorders and diseases in body systems such as the nervous, cardiovascular, and reproductive systems (1, 2). Lifestyle changes, modernization, and increased life expectancy have led to an increase in the age of marriage in developed and developing countries (3). The most prominent theory in relation to the aging process is the free radical theory, which states that the production of free radicals can lead to tissue and cellular oxidative damage and ultimately lead to aging and cell death. The anti-oxidant system cannot neutralize all free radicals produced during a lifetime therefore; oxidative stress causes various diseases and dysfunctions of the body systems (3, 4). 

Aging can impact the male reproductive system. Previous clinical studies have shown that aging reduces semen quality and increases sperm DNA fragmentation (5, 6). In this regard, U.S. statistics show that oxidative stress is one of the major causes of male infertility. 30 to 40% of infertile men have higher levels of ROS in plasma and semen (7). Aging reduces sex hormones secretion as well as the body’s anti-oxidant defense function and increases apoptosis and free radicals in the reproductive system (5, 7).

Since aging is a persistent and long process, the availability of aged animals may be a limiting factor for research (4). For this purpose, animal aging models have been developed including fruit flies, birds, fish, dogs, rats, and mice (8). It has been noted that D-galactose injection induces aging (8). D-gal-induced animals have shown aging-related changes in body physiology and phenotypes such as neurological deficit, decreased immune responses, activity of anti-oxidant enzymes, and increased ROS production and reproductive toxicity. According to the results of different studies, aging leads to degenerative changes in reproductive tissues along with a decrease in sperm quality and most matings do not lead to success in fertility (1, 3, 9). 

Due to the increase in the aging population and its effect on the reproductive system, it is essential to examine the factors that can have protective impacts on this process. The use of herbal medicines has increased globally due to fewer side effects, reasonable prices, and efficacy in most human diseases (10, 11). As potential factors for the production of new anti-aging drugs, herbal medicines have opened a new horizon and attracted a lot of attention (10). Medicinal plants, as the main source of natural anti-oxidants, are known to be beneficial for biological systems by protecting them against oxidative damage (10, 12). 

Caffeic acid (CA) is an organic and effective anti-oxidant compound that helps prevent the oxidation of molecules in the body. It is found naturally in a wide range of plants (especially in coffee) and has protective effects such as antiviral, anti-inflammation, anticancer, prevention of premature aging, reduction of exercise-induced fatigue, etc (13, 14). In this regard, previous studies have shown that CA can reduce the toxic effects of various factors such as cadmium (15), doxorubicin (16), and arsenic (17) on the reproductive system. The aim of this study is the investigation of the effects of CA on testicular damages in the D-galactose-induced aging model in mice.

## Materials and Methods

***Animals and study design***

In this experimental study, 40 adult male mice (approximately 2 months old) were used. The animals were procured from the Royan Institute, Iran and stored in standard conditions (22–24 °C with 12-hour light/dark cycle) in the animal house of Ardabil University of Medical Sciences, Iran. Animals had access to food and water freely during the study. All stages of work with animals and laboratory methods were carried out according to the protocols of the ethics committee of Ardabil University of Medical Sciences (IR.ARUMS.REC.1398.515). After acclimatization, the male mice were randomly divided into five groups (n=8) and treated as follows: 1) Control (con), 2) sham: equal volume of ethanol in normal saline was injected intraperitoneally once a day for 6 weeks, 3) Aging (Ag): D-galactose (300 mg/kg, Sigma-Aldrich) was injected intraperitoneally (IP) daily for 6 weeks, 4) Aging + caffeic acid (Ag+CA): plus D-galactose, caffeic acid (60 mg/kg, Sigma-Aldrich) was injected intraperitoneally (IP) daily for 6 weeks ( caffeic acid dissolved in a minimal volume of ethanol (99% pure) and then diluted in normal saline), and 5) caffeic acid (CA) (60 mg/kg) was injected intraperitoneally (IP) daily for 6 weeks. Dosages were selected based on previous studies (3, 18). 

***Sampling***

Twenty-four hours after the last injection, mice were weighed and then anesthetized (ketamine (50 mg/kg) and xylazine (10 mg/kg)) and the epididymis and testes were quickly separated. After weighing, one of the testicles was frozen in liquid nitrogen to investigate the oxidative stress factors including malondialdehyde (MDA), superoxide dismutase (SOD), glutathione peroxidase (GPx), and total anti-oxidant capacity (TAC) and the other testicle was fixed in 10% formalin to examine histopathological alterations. Furthermore, epididymis was used for sperm parameters evaluation. 

***Evaluation of sperm parameters***

The caudal epididymis of mice was used to evaluate sperm parameters. After isolation, the caudal part of the epididymis was placed in 2 ml of Ham’s F10 medium. Then it was sliced and placed in the CO_2_ incubator at 37 °C for 30 min. Next, the samples were diluted to 1:10, and 10 μl were placed on the Neobar slide and sperm parameters (count, morphology, motility, viability, and sperm DNA fragmentation (SDF)) were evaluated. Eosin-nigrosin staining was used to evaluate sperm viability and hematoxylin-eosin (H&E) staining was used for morphological evaluation (19, 20).

***Sperm DNA fragmentation measurement (SDF)***

The SDF kit (Ideh Varzan Farda, Iran) was used to measure sperm DNA fragmentation. After preparing the smears, 300 sperm were counted according to the kit protocol and the percentage of damaged sperm was calculated using the following formula (11): 

$\mathrm{SDF}\left(\%\right)=\frac{\mathrm{Fragmented}+\mathrm{Degraded}}{\mathrm{Total}\mathrm{sperm}\mathrm{count}}\times 100$

***Lipid peroxidation and anti-oxidant enzymes of testicular tissue***

At first, testicular tissue was homogenized in 1 ml KCL 1.15%. Lipid peroxidation (MDA levels) of tissue was measured using the ZellBio kit and the method described by KunioYagi (21). Anti-oxidant enzymes (SOD, GPx, and TAC) were also measured using the Ransel kit manufactured by the Randox kit (United Kingdom) based on the manufacturer’s protocol (11).

***Histopathological studies***

To investigate the histopathological changes, the fixated samples after tissue processing were embedded in paraffin (22). Then slides were prepared by random cutting with a microtome (5u) and stained by the H&E method. After preparation of the slides, histopathological changes were evaluated. Morphometric studies including measurement of the outer diameter and the germinal epithelium thickness were also evaluated. For this purpose, 5 sections of each sample and 10 seminiferous tubules were randomly examined according to Golmohammadi *et al*. (11) and Ameli *et al*. (23). Also, to evaluate the spermatogenesis index, Johnson’s score was calculated for seminiferous tubules. In addition, germinal epithelium cells were counted using the Image J software. 

***Statistical analysis***

All data (mean± standard error) were analyzed using Statistical package for the social sciences (SPSS) and one-way anova and Tukey tests. The results of *P*>0.05 were considered statistically significant.

## Results

***Effect of caffeic acid and aging on body weight and testicle***

Table 1 displays changes in body weight and testes of mice in different groups. The results showed that aging significantly reduces testicular weight in mice compared with the control group (*P*<0.05) but has no significant effect on body weight loss. Administration of CA as an anti-oxidant in the treated group showed that CA significantly prevents testicular weight loss (*P*<0.05). Also, the comparison between CA and Sham group with the control group did not show any significant change in the body and testes weight. 

***Effect of caffeic acid and aging on sperm parameters***

**Figure 1:**
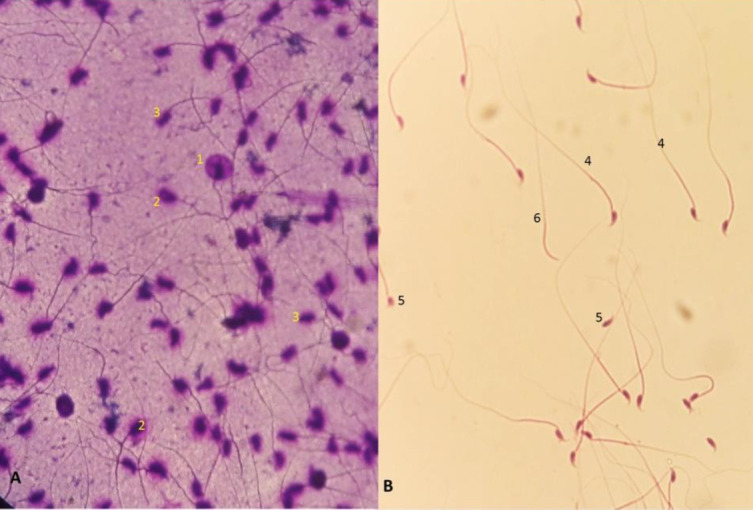
Representative of sperm in different staining methods. A: SDF staining shows normal (1) and DNA fragmented sperm (2 and 3). B: H&E staining shows normal (4), deformed head (5), and non-head (6) sperm

The results of this study showed that the number and viability of the sperms in the aging group significantly decreased compared with the control group (*P*<0.05). CA injection in the Ag+CA group significantly prevented this decrease in sperm count and viability (*P*<0.05). Sperm motility showed that progressive motility of sperm decreased significantly in the aging group (*P*<0.05). Statistical analysis showed that CA in the Ag+CA group significantly prevented sperm motility reduction compared with the Ag group (*P*<0.05). Sperm morphology (Figure 1) showed that the percentage of abnormal sperm in the aging group was significantly higher (*P*<0.05) than in the other groups (Table 2). In the Ag+CA group, the percentage of abnormal sperm decreased significantly compared with the aging group (*P*<0.05). Comparison between the caffeic acid and sham group with the control group did not show any significant change in sperm parameters (Table 2). 

***Effect of caffeic acid and aging on SDF***

The comparison of SDF showed that in the aging group, the percentage of SDF increased significantly compared with the control group (*P*>0.05). CA significantly decreased SDF percentage in the Ag+CA group compared with the aging group. The comparison between caffeic acid and sham group with the control group did not show any significant change (Table 2, Figure 1).

***Effect of caffeic acid and aging on lipid peroxidation of testicular tissue***

**Figure 2:**
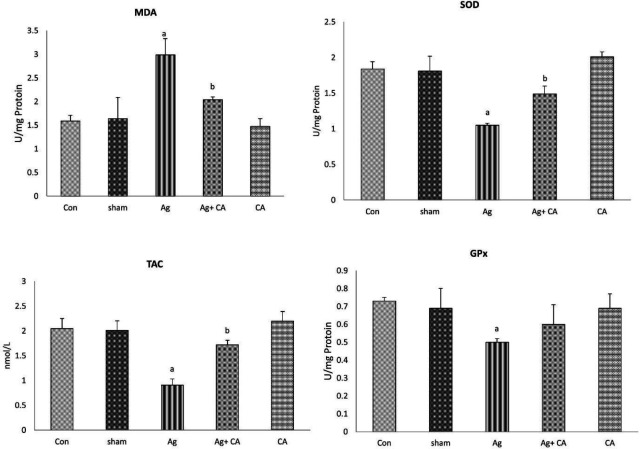
Comparing the level of Malondialdehyde (MDA), Superoxide dismutase (SOD), Glutathione peroxidase (GPx) and Total antioxidant capacity (TAC) in testicular tissue of different groups of study. Data are mean ± SE. a P<0.05 compared with control (con) and sham groups; b P<0.05 compared with Aging (Ag) group

The results of comparing the changes in testicular tissue MDA levels in the aging group showed a significant increase compared with the control group (*P*<0.05). CA administration in the Ag+CA group significantly reduced MDA levels compared with the aging group (*P*<0.05). Also, the comparison between the control group with the CA and sham group did not show any significant change in the level of MDA in testicular tissue (Figure 2). 

***Effect of caffeic acid and aging on the activity of GPx and SOD in testicular tissue***

The mean of GPx and SOD activity of testicular tissue in the aging group significantly decreased compared with the control group (*P*<0.05). CA administration in the Ag+ CA group significantly prevented the reduction of SOD activity (*P*<0.05) but had no significant effect on GPx (*P*=0.19). The comparison of GPx and SOD activity between the control group with caffeic acid and the sham group did not show any significant change (Figure 2). 

***Effect of caffeic acid and aging on TAC of testicular tissue***

The results showed that D-galactose-induced aging significantly reduced the total anti-oxidant capacity of the testis compared with the control group (*P*<0.05). CA administration in the treatment group significantly prevented the reduction of TAC compared with the aging group (*P*<0.05). Also, statistical analysis showed no significant difference between the control group with caffeic acid and the sham group (Figure 2). 

***Effect of caffeic acid and aging on histopathological changes in testicular tissue***

**Figure 3:**
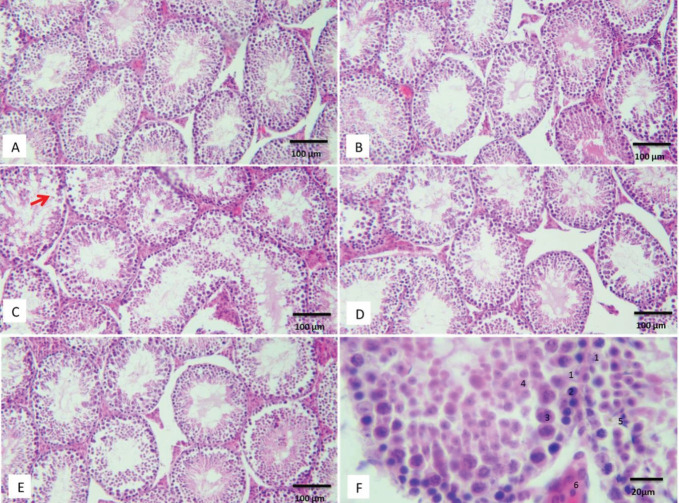
Representative photographs (H&E staining) of mice testes in con (A), sham (B), aging (C), Ag+CA (D), and CA groups (E). The red arrow indicates the destruction of the germinal epithelium. Type of counted cells (F) including Sertoli (1), spermatogonia (2), primary spermatocytes (3), spermatids (4), elongated spermatids (5) and Leydig (6)

Histological studies showed that seminiferous tubules were normal in control, sham, and caffeic acid groups (Figure 3). Observations showed that in the aging group, the germinal epithelium was degraded, and cell detachment was found in different parts (Figure 3). The spermatogenesis index showed that Johnson’s score in the aging group decreased significantly compared with the control group (*P*>0.05). Morphometric studies also showed that the outer diameter and the germinal epithelium thickness in the aging group had a significant decrease compared with the control group (*P*>0.05). CA administration in the treatment group (Ag+CA) prevented the destruction of testicular tissue and improved Johnson’s score and germinal epithelium thickness (*P*>0.05). Also, different cell counts showed that the number of germinal epithelium cells in the aging group was significantly reduced compared with the control group (*P*>0.05). CA treatment significantly prevented the reduction of germinal epithelium cells (*P*>0.05) (Figure 3 and Table 3). 

**Table 1:** Effects of caffeic acid on the body and testes weights in Dgalactose induced aging mice

Groups	Control	sham	Ag	Ag+CA	CA
Body weight at the beginning of the experiment (g)	28.61±1.74	27.93±2.43	27.72±1.64	29.58±1.30	29.80±1.52
Body weight at the end of the experiment (g)	38.55±1.13	37.23±1.56	36.07±1.43	38.52±1.08	38.86±1.47
Testes weight at the end of the experiment (mg)	131±0.09	130±0.13	93±0.10^a^	125±0.04^b^	136±0.03

Data are mean ± SE. a: *P*<0.05 compared with the control group; b: *P*<0.05 compared with the Aging group

**Table 2:** Effects of caffeic acid sperm parameters and SDF in Dgalactose induced aging mice

Groups	Control	sham	Ag	Ag+CA	CA
Sperm count (106/mL)	15.43±1.56	15.13±1.19	10.02±1.46^a^	13.65±3.84^b^	15.97±1.58
Sperm viability (%)	86.0±2.02	85.25±1.81	43.34±1.93^a^	58.62±1.32^b^	87.87±1.56
Rapid progressive motility (%)	50.24±2.1	49.91±0.95	35.16±1.7^a^	44.38±1.32^b^	52.02±1.38
Slow progressive motility (%)	22.74±2.26	21.96±1.91	14.74±1.08^a^	19.53±2.78	23.91±1.57
In situ motility (%)	14.39±1.37	14.86±0.86	17.73±1.90	14.56±2.1	13.97±1.37
Non-motile (%)	14.48±1.73	14.02±0.87	34.65±2.27^a^	20.47±1.62^b^	12.41±1.51
Normal sperm (%)	89.87±3.44	88.12±1.12	68.25±4.97^a^	79.87±3.56^b^	89.75±1.85
Abnormal sperm (%)	10.12±1.21	11.87±0.39	31.75±1.76^a^	20.12±1.22^b^	10.25±0.69
SDF (%)	16.0±1.58	16.37±1.52	58.37±1.51^a^	42.25±3.17^b^	14.12±1.61

Data are mean ± SE. a: *P*<0.05 compared with the control group; b: *P*<0.05 compared with the Aging group

**Table 3:** Effects of caffeic acid and aging on histopathological changes of testicular tissue

Parameters		Control	Sham	Ag	Ag + CA	CA
Outer diameter (lm)		214.62±2.68	212.46±1.29	146.59±4.79^a^	169.13±6.62^b^	220.98±3.58
Thickness of germinal epithelium (lm)		81.14±3.14	79.00±3.26	54.84±3.35^a^	70.58±1.84^b^	82.93±2.75
Mean Johnsen score (MJS)		8.75±0.70	8.37±0.74	5.12±1.24^a^	6.62±1.18^b^	8.87±0.74
Spermatogonia number		60.47±1.15	59.32±.3.02	56.01±1.54	58.14±4.01	60.47±1.54
primary spermatocytes number		58.87±1.35	57.72±.2.02	47.97±3.74^ a^	50.14±2.11	59.90±0.54
Spermatids number		78.57±4.31	76.12±.3.22	58.99±1.74^ a^	64.45±3.41^ b^	78.71±2.34
elongated spermatids number		80.91±4.11	77.77±.3.18	53.48±2.41^ a^	63.34±1.99^ b^	81.97±3.48

Data are mean ± SE. a: *P*<0.05 compared with the control group; b:* P*<0.05 compared with the Aging group

## Discussion

This study investigated the effects of CA on weight, oxidative stress factors, sperm parameters, and histopathological changes of testis in the D-galactose-induced aging model in mice.

In the present study, evaluation of body weight and testis of mice showed that aging did not cause significant changes in the body weight of mice, but it could significantly reduce testicular weight. These results are consistent with previous studies on the effect of aging on body weight and sexual organs. In this regard, Mohammadi *et al*. have shown that aging reduces the weight of sexual organs (3, 10). One of the reasons for testicular weight loss due to aging is the destruction of seminiferous tubules; our study also showed degenerative changes in testicular tissue. 

According to the results of different studies, increasing rodent age causes degrading changes in testicle tissue along with a decrease in sperm quality (3, 4, 10). Treatment of aging mice with CA prevented testicular weight loss. Benallah Abd *et al*. have shown that CA prevents body weight loss and sexual organs in mice treated with lambda Cyhalothrin (24). This protective action of CA can be due to its anti-oxidant properties. because previous studies have shown that administration of anti-oxidants can reduce the effects of aging on the weight of sexual organs (3, 9). 

Also, the results of this study showed that D-galactose-induced aging causes a reduction in sperm count, motility, and viability, as well as an increase in abnormal sperm and SDF. One of the factors affecting these parameters is the decrease in sex hormone levels and increased oxidative stress. Studies have shown that aging reduces sex hormones and increases oxidative stress, which can cause spermiogenesis disorder and consequently a significant decrease in sperm parameters (1, 4, 7).

Due to the considerable amount of fatty acids in the cell membrane of the sperm, it is highly sensitive to damage caused by free radicals and lipid peroxidation (LPO) (11, 20). LPO reduces sperm viability and increases morphological defects, which can also lead to a decrease in sperm viability and motility (5). Salimnejad *et al*. have shown that oxidative stress increases SDF in mice (20). In the present study, it was observed that D-galactose-induced aging increases SDF. Since D-galactose induces the aging model by induction of oxidative stress, it is assumed that sperm parameter disruption can be due to D-galactose-induced oxidative stress. In this regard, the results of this study showed that D-galactose-induced aging significantly increases the level of testicular tissue MDA (as a lipid peroxidation marker) compared with the control group. This finding is consistent with the results of previous studies which have also stated that aging increases MDA levels (9, 25). 

Sperm loses a major volume of cytoplasm during spermiogenesis and its anti-oxidant enzyme concentration decreases, so it is more sensitive to oxidative damage than any other cell (20). Our results showed that D-galactose-induced aging reduces the activity of GPx, SOD, and TAC compared with the control group, which is consistent with the results of previous studies (3, 10, 25). GPx, as an anti-oxidant by being located in the plasma membranes of sperm, sperm nucleus, and semen, protects sperm against free radicals and contributes to the final maturation of sperm (20). SOD also prevents cell damage by preventing the formation of superoxide free radicals. A decrease in the activity of this enzyme due to aging can disrupt spermatogenesis and damage testicular tissue (20). Histological investigations in our study showed that aging caused degenerative changes in seminiferous tubules. Johnson’s score and germinal epithelium thickness also decreased in this group. These damages can be caused by oxidative stress. Taito Nakano *et al*. have shown that aging can cause degradation of germinal epithelium and impaired spermatogenesis (26). 

Previous studies have shown that the use of anti-oxidants can reduce the effects of aging on the reproductive system. In this regard, Hamza *et al*. have shown that the administration of resveratrol and quercetin can improve reproductive parameters in those D-galactose-induced aging mice (1). Mohammadi *et al*. have also shown that curcumin administration can reduce the effects of aging on the male reproductive system by reducing oxidative stress and apoptotic cells (3). 

In our study, the results showed that administration of CA as an anti-oxidant reduces the effects of aging on sperm parameters. This finding is consistent with the results of Abdallah *et al*. (2014). They have also described that administration of CA improves sperm parameters in rats treated with lambda-cyhalothrin (24). CA may improve sperm parameters by reducing oxidative stress. Studies showed that CA administration reduces lipid peroxidation and increases anti-oxidant enzyme activity in testicular tissue, which confirms the role of CA in reducing oxidative stress (13, 18). Researchers have also detected that CA can reduce lipid peroxidation and increase the activity of anti-oxidant enzymes in testicular tissue against arsenic-induced damage (17). Refaei *et al*. have also shown that CA reduces testicular damage caused by cadmium by improving the activity of anti-oxidant enzymes (15). Histopathological results of the present study also revealed that treatment with CA prevents testicular damage and germinal epithelium thickness and Johnson score. This finding is in line with previous studies which have also stated that CA can prevent testicular tissue from various damages by reducing lipid peroxidation (15, 17, 24).

## Conclusion

The results of this study show that CA as an anti-oxidant can inhibit lipid peroxidation and increase the anti-oxidant defense system to prevent testicular tissue damage against D-galactose-induced aging and thus improve sperm parameters. It is recommended that in future studies the effects of different doses of CA on other fertility parameters be investigated. 

## Authors’ Contributions

FK Designed and performed the experiments. MGG Verified the analytical methods. MJD Wrote the manuscript. NN Wrote the manuscript. RS Developed the theory and performed the computations. 

## Funding Source

We extend our thanks to Ardabil University of Medical Sciences, Iran, for their financial support. 

## Conflicts of Interest

The authors declare that they have no conflicts of interest.
